# Supplementary material for: Effect of BIO-PLYTM, a Platelet-Rich Plasma Derived Biologic on PRRSV-2-Infected Macrophages
Source: Viruses. 2022 Nov 28;14(12):2666. doi: 10.3390/v14122666 (PMC9783555; doi:10.3390/v14122666)
Supplement: Supplementary file 1 [file viruses-14-02666-s001.zip › viruses-2027402-Supplementary.pdf]

Supplementary Figures

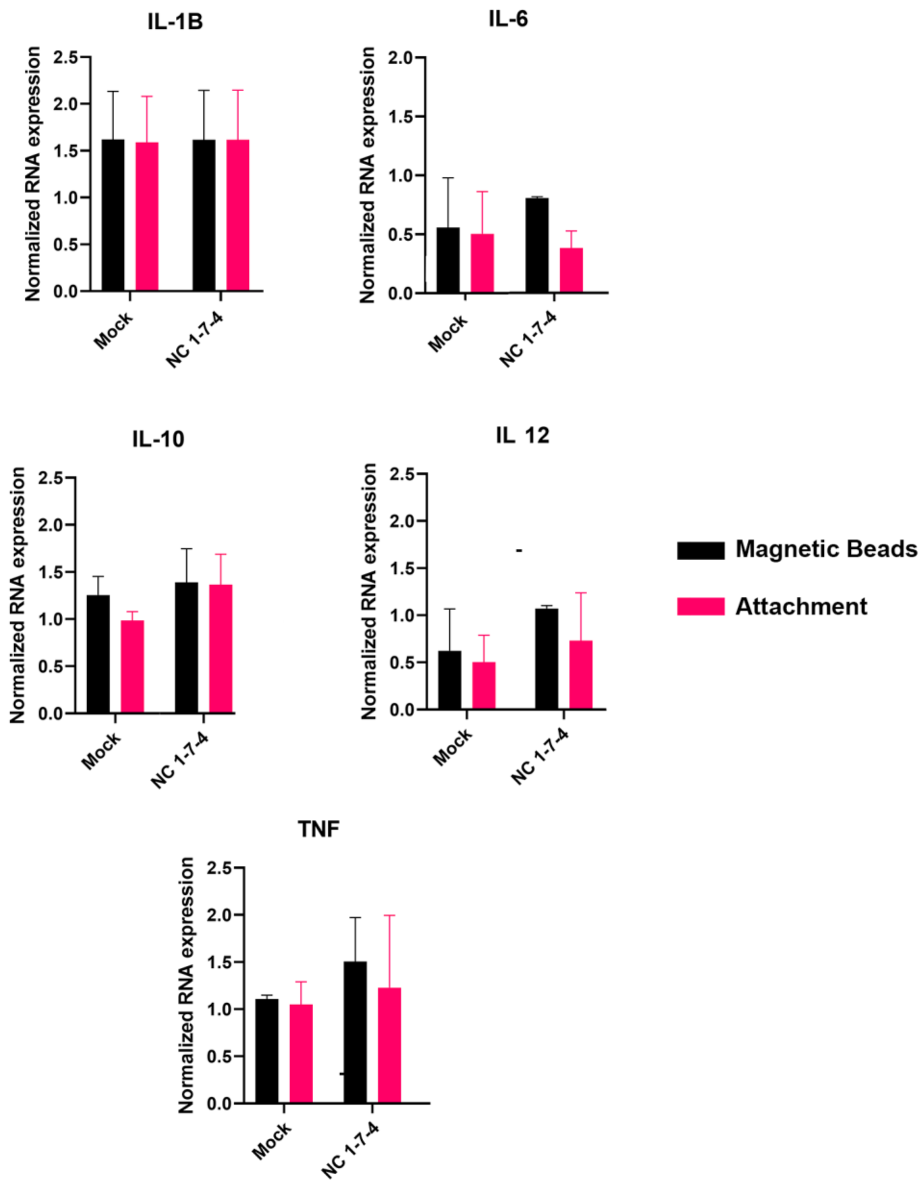

Figure S1: Comparison of the cytokine responses of macrophages isolated using magnetic beads or attachment to plastic cultures plates

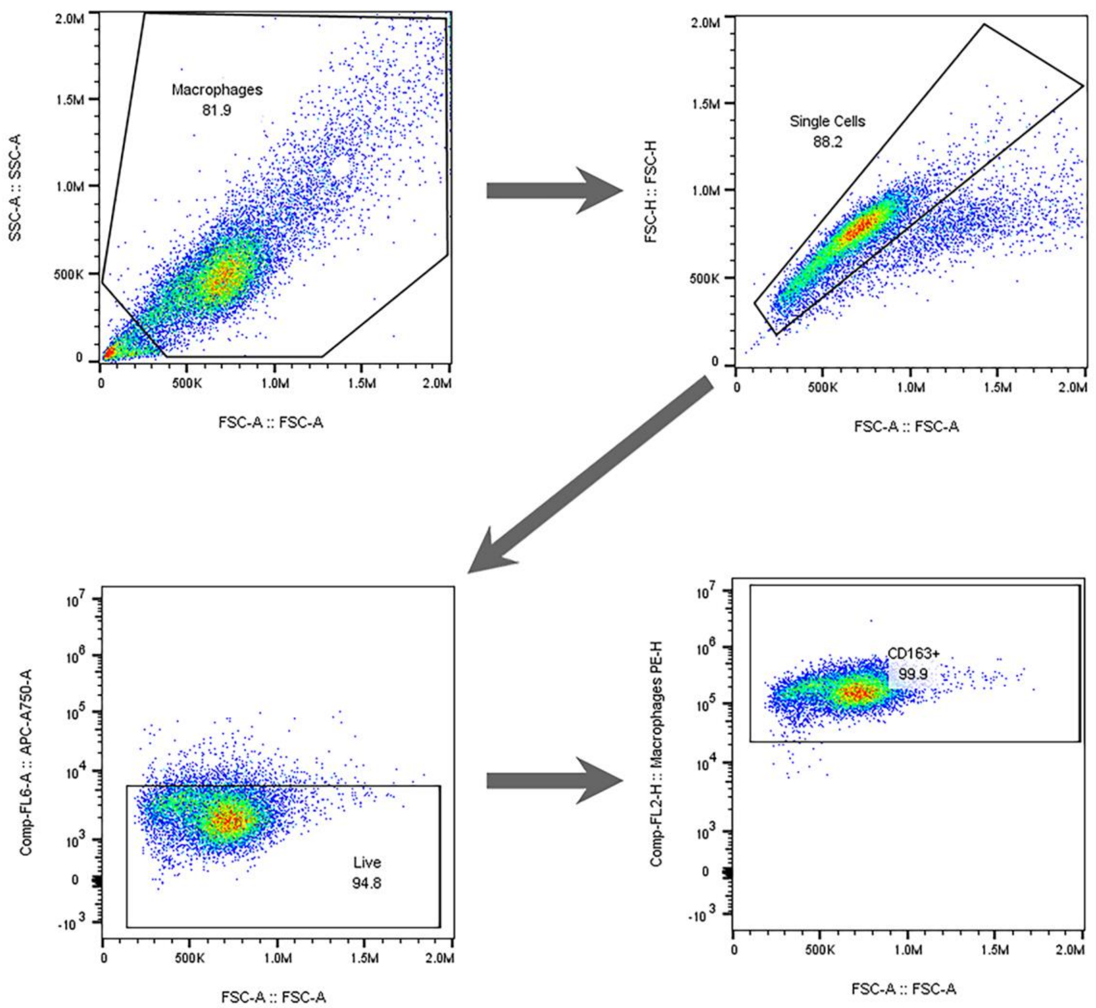

Figure S2: Gating strategy for the macrophage viability assay

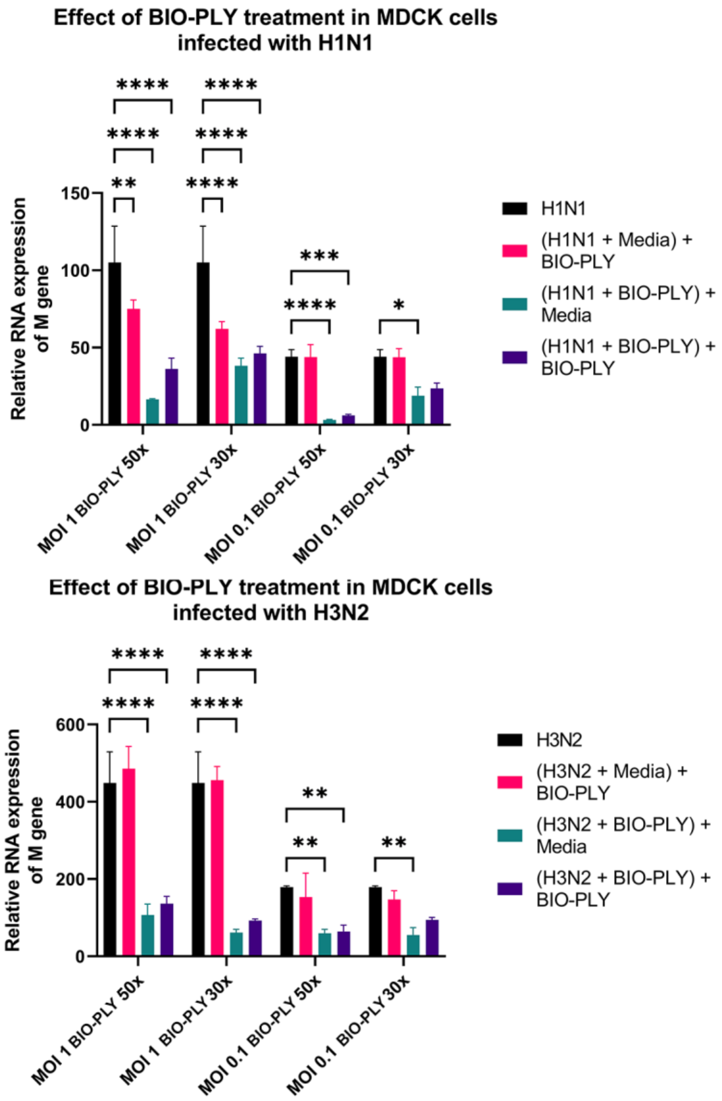

Figure S3: Effect of BIO-PLY™ on the reduction of viral copies in MDCK cells infected with human influenza

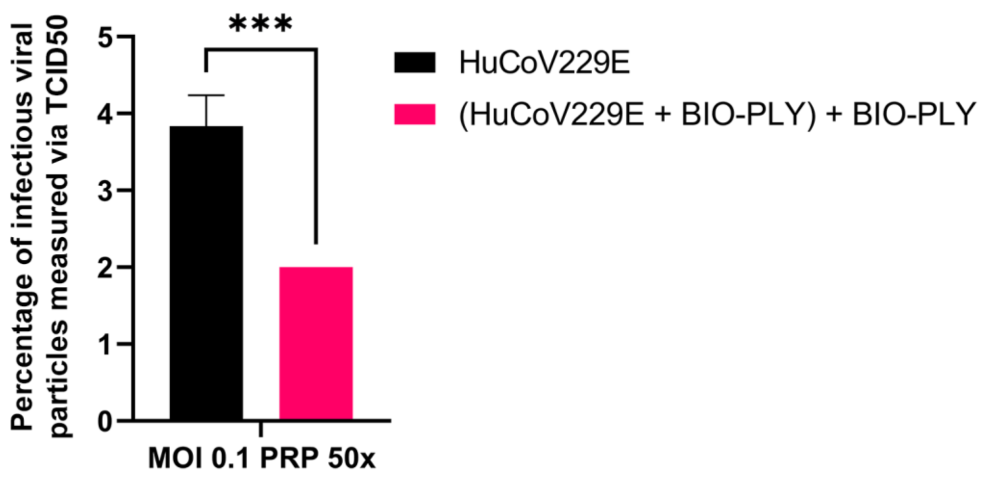

Figure S4: Effect of BIO-PLY™ on the reduction of human coronavirus HuCoV229E titer in Huh-7 cells

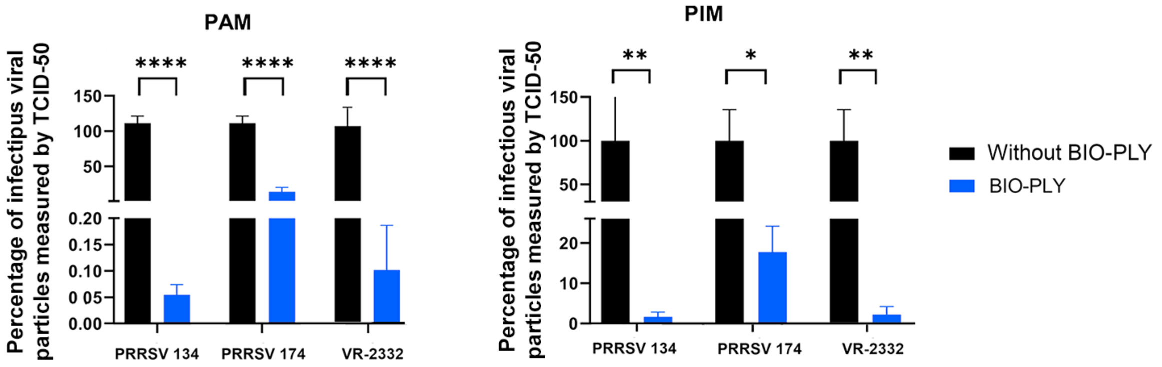

Figure S5: Effect of equine BIO-PLY™ treatment on the production of infectious viral particles in porcine alveolar macrophages (PAM) and pulmonary intravascular macrophages (PIM) measured as TCID50.
